# Supplementary material for: Western Hognose Snakes (Heterodon nasicus) Prefer Environmental Enrichment
Source: Animals (Basel). 2022 Nov 29;12(23):3347. doi: 10.3390/ani12233347 (PMC9739432; doi:10.3390/ani12233347)

Supplementary Information

Table S1: Bayesian repeated measures ANOVA results table. The table gives the Bayes Factor (BF) for each model compared to the null, and the Bayesian inclusion factor (BF_{incl}) for each main effect or interaction.

**Model**	**BF**	**BF_{incl}**
Day	3.513	3.555
Hot	1.367	1.389
Day + Hot	4.903	
Day + Hot + Day*Hot	0.412	0.084

Figure S1: the preference testing arena. Top panel: a schematic of the preference testing arena. Bottom panel: a photograph of one preference testing cage.

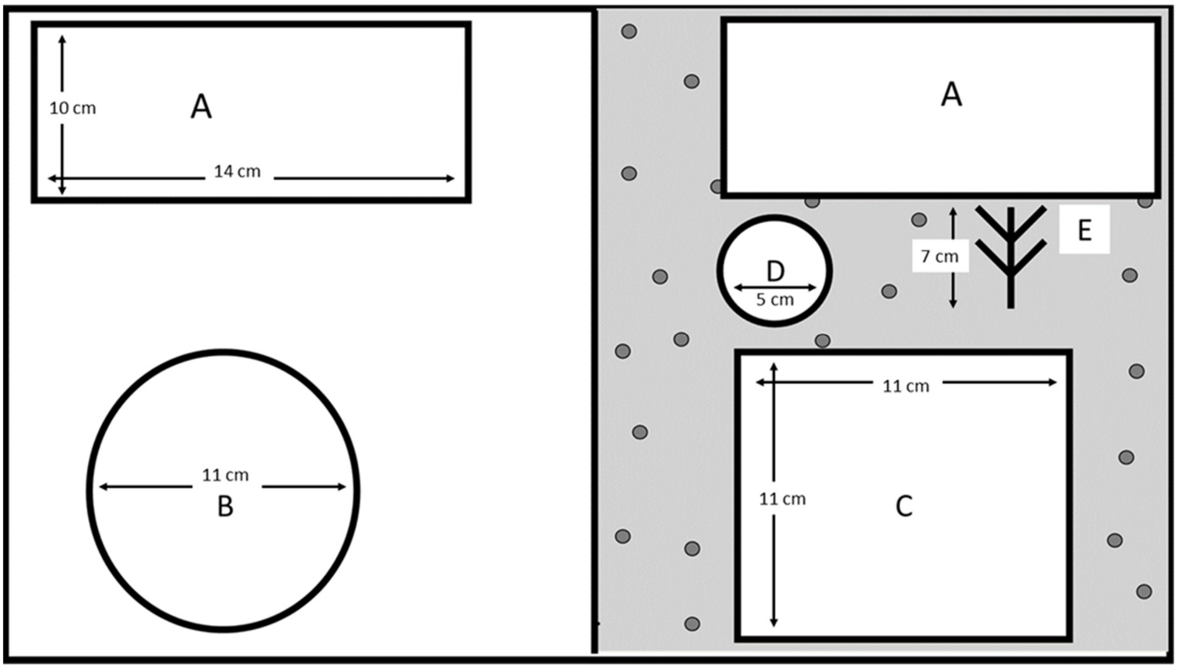

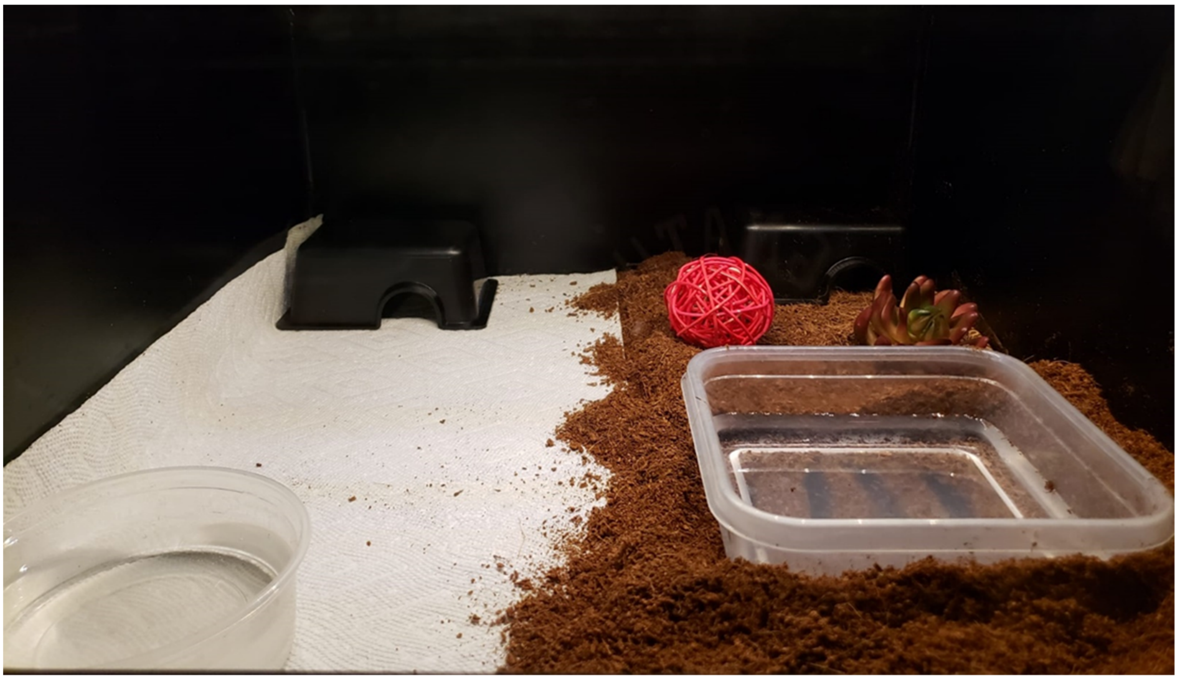

Figure S2: Photo of the boldness assay arena.

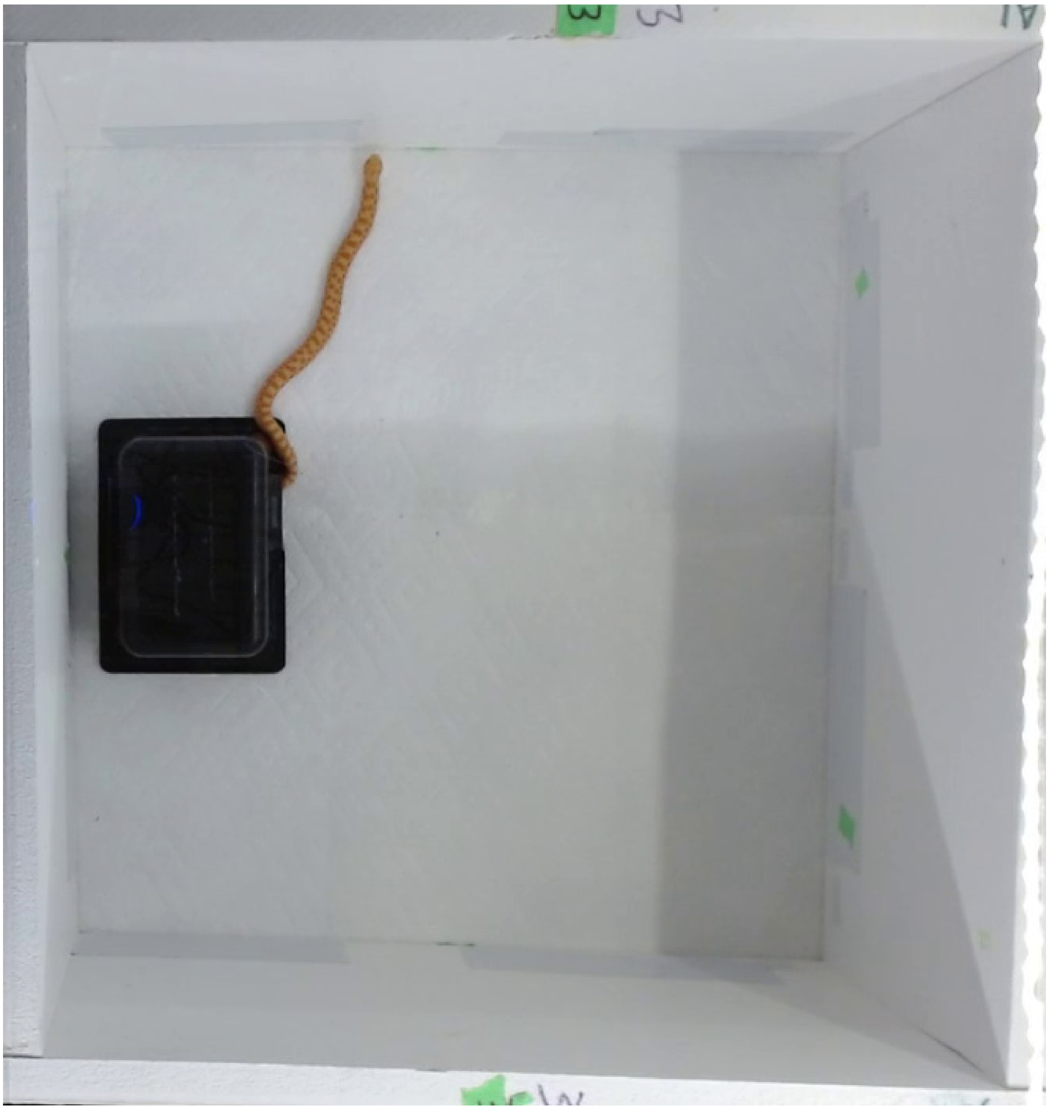

Figure S3: Graph indicating the thermal gradient within enclosures.

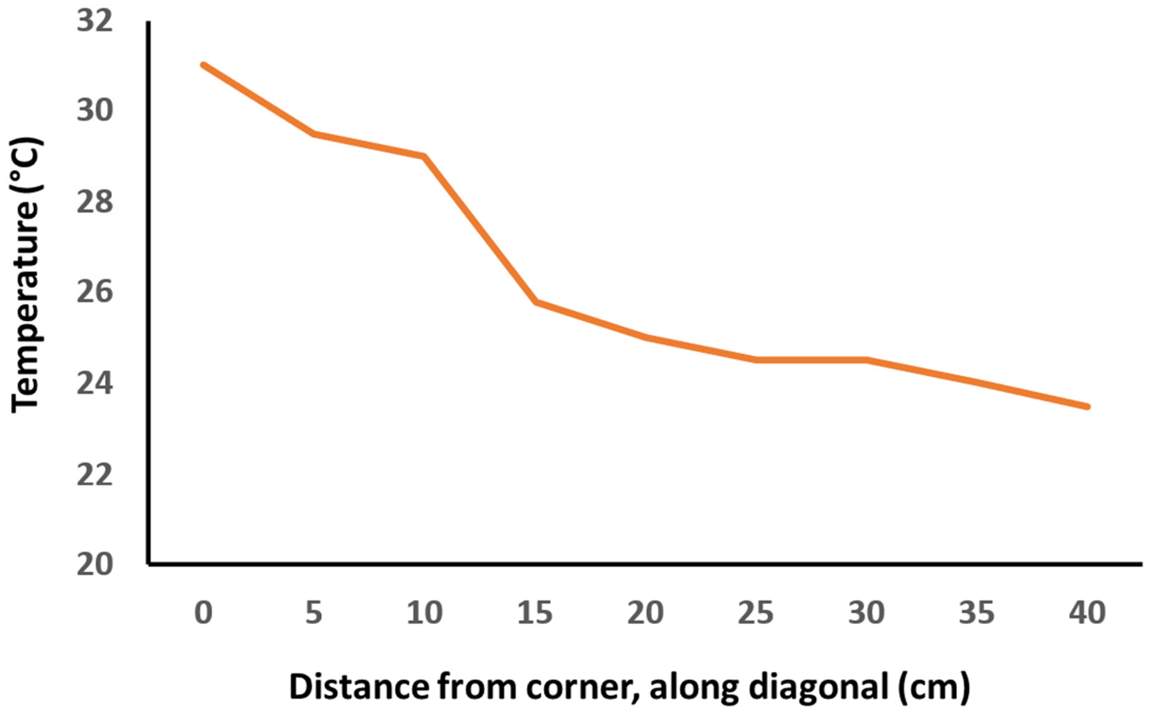

Supplement: Supplementary file 1 [file animals-12-03347-s001.zip › animals-2024780-supplementary.pdf]
